# The Gull Alpha Power Lomax distributions: Properties, simulation, and applications to modeling COVID-19 mortality rates

**DOI:** 10.1371/journal.pone.0283308

**Published:** 2023-09-07

**Authors:** Ahlam H. Tolba, Abdisalam Hassan Muse, Aisha Fayomi, Hanan M. Baaqeel, Ehab M. Almetwally

**Affiliations:** 1 Mathematics Department, Faculty of Science, Mansoura University, Mansoura, Egypt; 2 Faculty of Science and Humanities, School of Postgraduate Studies and Research, Amoud University, Borama, Somalia; 3 Faculty of Science Department of Statistics King Abdulaziz University, Jeddah, Saudi Arabia; 4 Department of Statistics, Faculty of Business Administration, Delta University for Science and Technology, Gamasa, Egypt; Iowa State University, UNITED STATES

## Abstract

The Gull Alpha Power Lomax distribution is a new extension of the Lomax distribution that we developed in this paper (GAPL). The proposed distribution’s appropriateness stems from its usefulness to model both monotonic and non-monotonic hazard rate functions, which are widely used in reliability engineering and survival analysis. In addition to their special cases, many statistical features were determined. The maximum likelihood method is used to estimate the model’s unknown parameters. Furthermore, the proposed distribution’s usefulness is demonstrated using two medical data sets dealing with COVID-19 patients’ mortality rates, as well as extensive simulated data applied to assess the performance of the estimators of the proposed distribution.

## 1 Introduction

Researchers have been contributing to the theory of probability in recent years in order to overcome some of the limitations of statistical models. The Exponential distribution, for example, cannot handle data characterized by hazard rates that are monotonic or non-monotonic; it can only describe an object’s constant hazard rate. The gamma distribution has the shortcoming that it can only handle data with an increasing failure rate. However, real-life data is characterized by a non-monotonic hazard rate function. In distribution theory, efforts will always be made to generalize distributions. The essence of generalizing distributions is to obtain more robust and flexible models that have a wide range of applications. To achieve this, many methods are applied, as revealed by numerous pieces of literature. Also, the analysis and empirical results obtained greatly depend on how appropriately the chosen distribution fits the data under consideration.

Modifying current probability models to handle hazard rates that are both monotonic and non-monotonic, as well as provide an acceptable fit, is common practice. The new family of distributions was developed by [1] by using the Logit function and studying the Gumbel-Weibull distribution. [2, 3] studied the gamma-X family of distributions and the normal distribution as special cases of the model. For more reading on the developed distributions and their applications in reliability analysis, medical research, and the biological situation, there may be more than one cause of failure competing for the event. The event can be either death or recovery from a certain disease (risk), as referred to [4] introduced a competing risk model with lifetime Weibull sub-distributions, [5] studied statistical inference to the parameter of the Akshaya distribution under competing risk data with the application of HIV infection to AIDS; and [6] discussed statistical analysis of a regression competing risk model with covariates using Weibull sub-distributions. Also, [7] discussed the analysis of the Thymic Lymphoma of Mice application and estimation for the Akshaya failure model with competing risks, and [8] presented the conclusions for the stress-strength reliability model with a partially accelerated life test for its strength variable. More applications for developed distributions have been discussed by [9–25].

The goal of creating a new distribution family is to create a new statistical model in order to solve some of the problems with existing probability distributions. Not only will the proposed distribution handle different types of hazards, but it will also increase flexibility and produce a better fit than alternative probability models. In the literature, there are distributions. In this study, the Gull Alpha power family of distributions is proposed as a novel family of distributions. distribution. The Lomax distribution is used to derive the specific case of this family. Gull Alpha Power Lomax Distribution is also known as Gull Alpha Power Lomax Distribution (GAPL). The GAPL distribution is a modified version of the Lomax distribution that can be used to simulate non-monotonic hazard rate shapes. The hazard function, survival function, and moments of the distribution have been derived. Application to real data sets to demonstrate the versatility of the proposed model is done.

A new family of distribution called the Gull Alpha Power Family (GAPF) was developed by [26]. The CDF and the PDF of the family are given as follows:

$$\begin{array}{c} F_{GAPF}\left(x\right)=\{\begin{array}{cc} \frac{\alpha F(x)}{\alpha^{F(x)}} & \;\text{if}\;\alpha >0,\\ F(x)=F(x) & \;\text{if}\;\alpha =1, \end{array} \end{array}$$ (1)

where *α* is the shape parameter. The PDF of the family is:

$$\begin{array}{c} f_{GAPF}\left(x\right)=\{\begin{array}{cc} \text{log}\left(\alpha \right)\alpha^{1-F(x)}(-f(x)F(x))+f\left(x\right)\alpha^{1-F(x)} & \;\text{if}\;\alpha >0,\\ F(x)=F(x) & \;\text{if}\;\alpha =1, \end{array} \end{array}$$ (2)

This article is arranged as follows: In Section 2, we present and describe the Gull Alpha power Lomax distribution (GAPL), and its mathematical characteristics are presented in Section 3. Section 4 gives detailed estimation methods such as maximum likelihood, confidence intervals, bootstrap-p, and bootstrap-t for the unknown parameters. Bayesian analysis is discussed in Section 5. The numerical computations were performed to assess the behavior of estimates in Section 6. Also, In Section 7, we present two Applications to COVID-19 data sets. Finally, concluding remarks are mentioned in Section 8.

## 2 Gull Alpha Power Lomax Distribution (GAPL)

The CDF of the Lomax distribution is used to explain the specific form of GAPF in this section. The [27], also known as Pareto II, distribution has been frequently used in a variety of situations. [28] discussed moments of dual generalized order statistics and characterization for the transmuted exponential model. [29] obtained order statistics of inverse Pareto distribution. The Lomax distribution has been used for reliability modeling and life testing (e.g., [30]), and applied to income and wealth distribution data ([31, 32]), firm size ([33]), and queuing problems ([31, 32]). It’s also been used in the biological sciences, and it’s even been used to estimate the distribution of file sizes on servers ([34]). When the data is heavy-tailed, some authors, such as [35], have advised using this distribution instead of the exponential distribution. The Lomax distribution can be motivated in a number of ways. For example, [36]) show that it arises as the limit distribution of residual lifetime at a great age, and [37] studied the relates of the Lomax distribution to the Burr family of distributions. On the other hand, the Lomax distribution has been used as the basis for several generalizations. For example, [38] extend it by introducing an additional parameter using the [39] approach; [40] use the Lomax distribution as a mixing distribution for the Poisson parameter and derive a discrete Poisson-Lomax distribution, and [41] introduced the double-Lomax distribution and applied it to IQ data. The record statistics of the Lomax distribution have been studied by [42] the implications of various forms of right-truncation and right-censoring are discussed by [3, 43] and others; and sample size estimation has been discussed by [44].

The CDF of the Lomax distribution [44] is given by

$$\begin{array}{c} F_{L}\left(x\right)=1-(1+\frac{x}{\lambda }{)}^{-\theta } \end{array}$$ (3)

and the probability density function

$$\begin{array}{c} f_{L}\left(x\right)=\frac{\theta }{\lambda }(1+\frac{x}{\lambda }{)}^{-(\theta +1)},\theta ,\lambda ,x>0, \end{array}$$ (4)

where *θ* and λ are the shape and scale parameters, respectively.

The CDF and PDF of the GAPL distribution are given, respectively:

$$\begin{array}{c} F_{GAPL}\left(x\right)=\frac{\alpha \left(1-{\left(1+\frac{x}{\lambda }\right)}^{-\theta }\right)}{\alpha^{\left(1-{\left(1+\frac{x}{\lambda }\right)}^{-\theta }\right)}},\theta ,\alpha ,\lambda ,x>0 \end{array}$$ (5)

and the probability density function

$$\begin{array}{c} f_{GAPL}\left(x\right)=\frac{\theta }{\lambda }(1+\frac{x}{\lambda }{)}^{-(\theta +1)}\alpha^{{\left(1+\frac{x}{\lambda }\right)}^{-}\theta }[1-\text{log}\;\alpha (1-{\left(1+\frac{x}{\lambda }\right)}^{-\theta })], \end{array}$$ (6)

where *θ*, *α* and λ are greater than zero.

**Fig 1:**
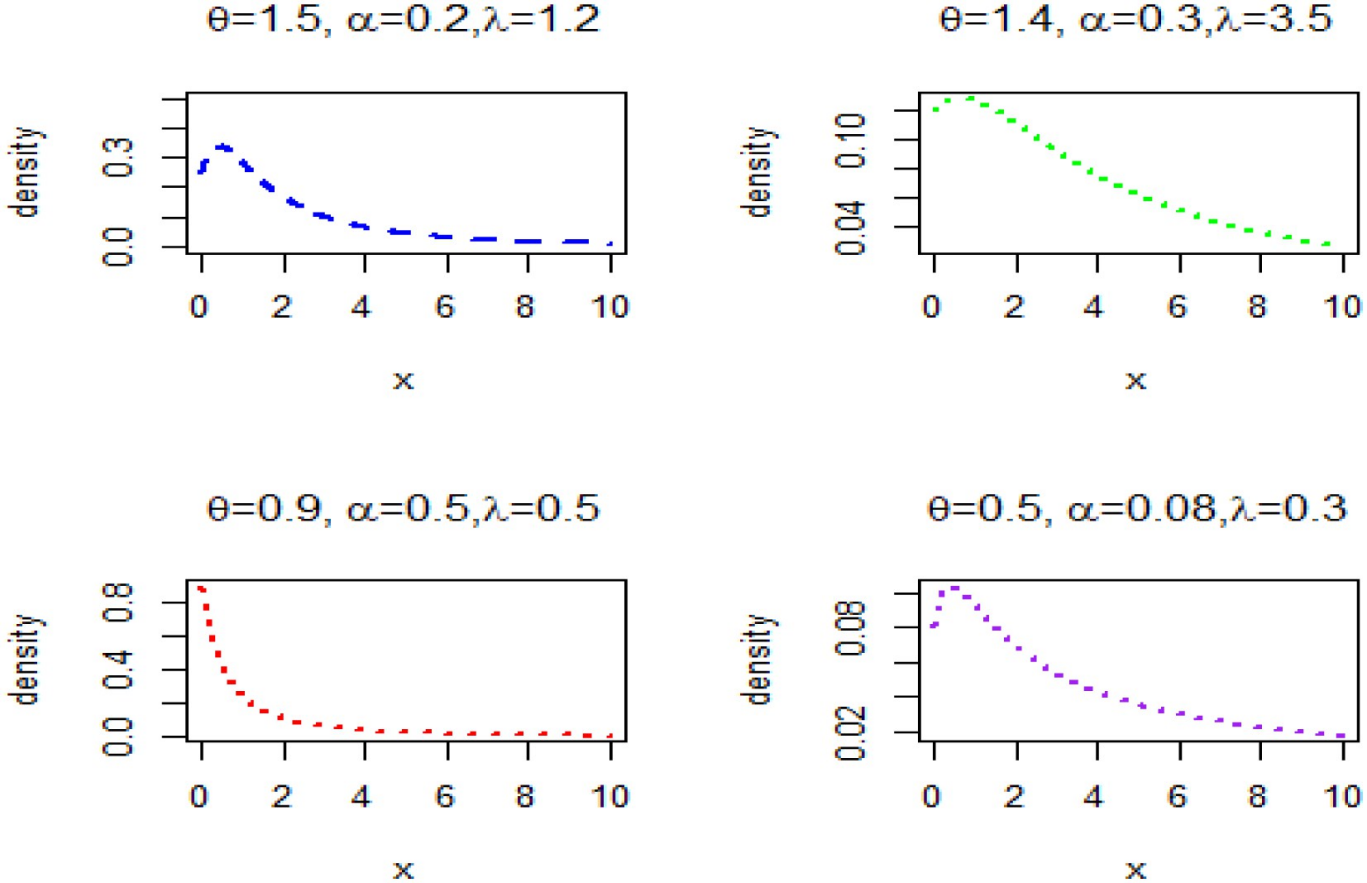
Plots of PDF for different parameter values.

The GAPL distribution is characterized by three parameters *α*, *θ*, λ. The PDF graphical representations for a different set of parameter values are given in Fig 1.

### 2.1 Hazard and survival functions

The hazard and survival functions for the GAPL distribution are defined in this section.

$$\begin{array}{c} S_{GAPL}\left(x\right)=1-\frac{\alpha (1-(1+\frac{x}{\lambda }{)}^{-\theta })}{\alpha^{\left(1-{\left(1+\frac{x}{\lambda }\right)}^{-\theta }\right)}}, \end{array}$$ (7)

and

$$\begin{array}{c} H_{GAPL}\left(x\right)=\frac{\frac{\theta }{\lambda }(1+\frac{x}{\lambda }{)}^{-(\theta +1)}\alpha^{{\left(1+\frac{x}{\lambda }\right)}^{-}\theta }[1-\text{log}\;\alpha \left(1-{\left(1+\frac{x}{\lambda }\right)}^{-\theta }\right)]}{1-\frac{\alpha \left(1-{\left(1+\frac{x}{\lambda }\right)}^{-\theta }\right)}{\alpha^{\left(1-{\left(1+\frac{x}{\lambda }\right)}^{-\theta }\right)}}}, \end{array}$$

where *θ*, *α* and λ > 0.

**Fig 2:**
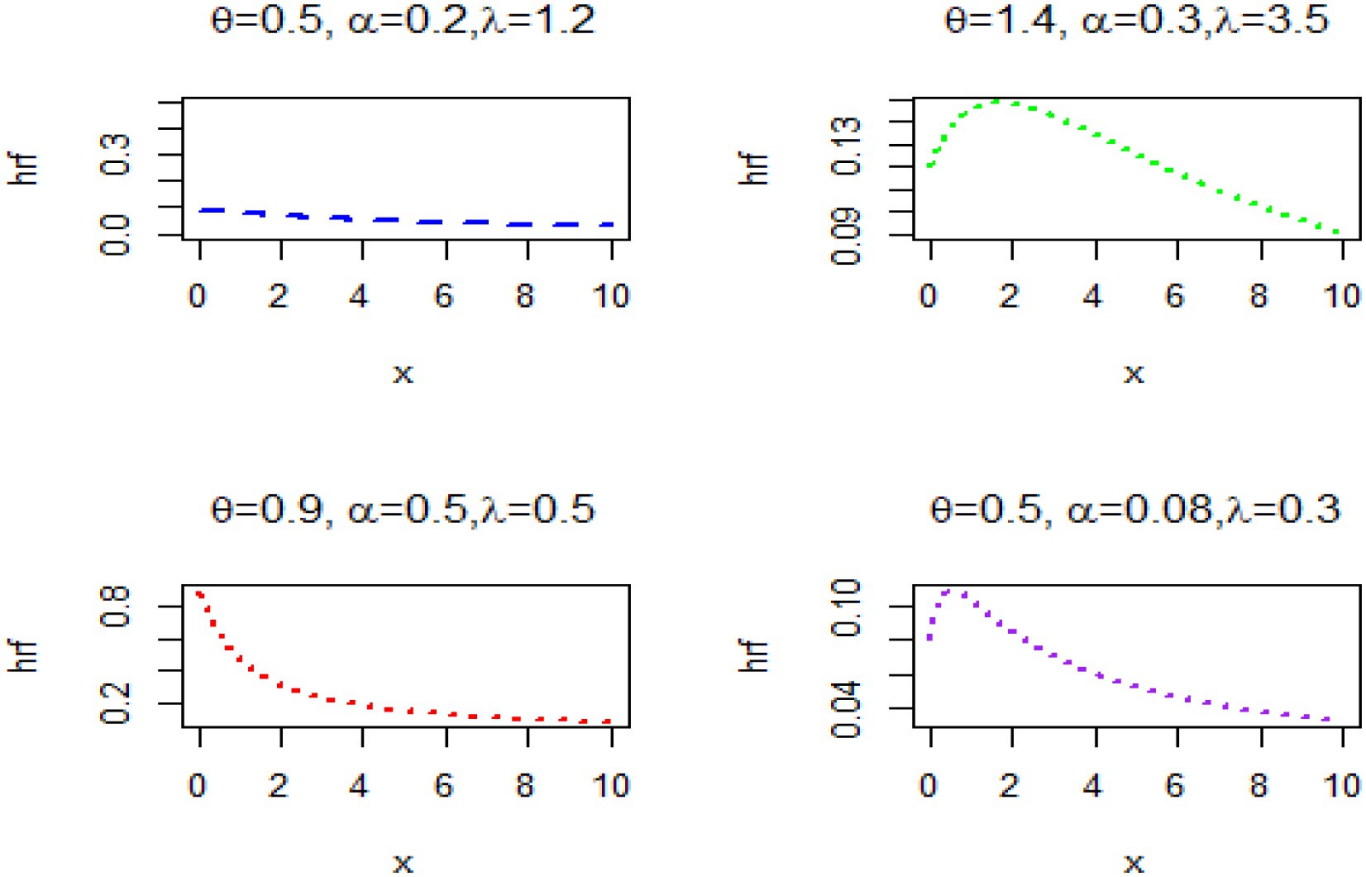
Plots of HR for selected parameter values.

The hazard rate plot is displayed in Fig 2.

## 3 Statistical properties

In this section, some important statistical properties have been discussed.

### 3.1 Quantile function

The importance of the quantile function is to get quantiles and assist in the simulation study. The quantile function is given as:

$$\begin{array}{c} F(x)=\mu \end{array}$$

$$\begin{array}{c} \frac{\alpha \left(1-{\left(1+\frac{x}{\lambda }\right)}^{-\theta }\right)}{\alpha^{\left(1-{\left(1+\frac{x}{\lambda }\right)}^{-\theta }\right)}}=u \end{array}$$ (8)

$$\begin{array}{c} x=\lambda \left(-1+{\left(\frac{\text{log}\;\alpha }{\text{log}\;\alpha +W_{-1}\left(\frac{-u\;\text{log}\;\alpha }{\alpha }\right)}\right)}^{\frac{1}{\theta }}\right). \end{array}$$ (9)

For the median, put u = 0.5 in Eq (9). Table 1 gives the quantiles for specified parameter values.

**Table 1:** The quantiles of the GAPL distribution for some values of the parameters.

Quantiles	λ = 0.3, *θ* = 0.4, *α* = 0.5	λ = 1.3, *θ* = 0.8, *α* = 0.7	λ = 0.3, *θ* = 1.8, *α* = 0.2
0.1	0.0243	0.1614	0.2785
0.2	0.0501	0.3548	0.6394
0.3	0.0788	0.5932	1.1611
0.4	0.1119	0.8979	1.9677
0.5	0.1520	1.3053	3.3102
0.6	0.2032	1.8868	5.7763
0.7	0.2741	2.7992	11.0342
0.8	0.3856	4.4875	25.5636
0.9	0.6178	8.9757	97.9262

### 3.2 Moments

The *r*^*th*^ moments of the GAPL distribution are defined as

$$\begin{array}{c} E\left(x^{r}\right)=\int_{0}^{\infty }x^{r}f\left(x\right)dx, \end{array}$$

$$\begin{array}{c} E\left(x^{r}\right)=\int_{0}^{\infty }x^{r}\frac{\theta }{\lambda }{\left(1+\frac{x}{\lambda }\right)}^{-\left(\theta +1\right)}\alpha^{{\left(1+\frac{x}{\lambda }\right)}^{-}\theta }[1-\text{log}\;\alpha \left(1-{\left(1+\frac{x}{\lambda }\right)}^{-\theta }\right)]dx. \end{array}$$ (10)

### 3.3 Order statistics

For an ordered random sample *X*_1_, *X*_2_, ………, *X*_*n*_ from the GAPL distribution the PDF of the *i*^*th*^ minimum and maximum order statistic is given by

$$\begin{array}{c} f_{X\left(1\right)}\left(x\right)=n\frac{\theta }{\lambda }{\left(1+\frac{x}{\lambda }\right)}^{-\left(\theta +1\right)}\alpha^{{\left(1+\frac{x}{\lambda }\right)}^{-}\theta }[1-\text{log}\;\alpha \left(1-{\left(1+\frac{x}{\lambda }\right)}^{-\theta }\right)]\\ {\left(1-\frac{\alpha \left(1-{\left(1+\frac{x}{\lambda }\right)}^{-\theta }\right)}{\alpha^{\left(1-{\left(1+\frac{x}{\lambda }\right)}^{-\theta }\right)}}\right)}^{n-1}, \end{array}$$ (11)

and

$$\begin{array}{c} f_{X\left(n\right)}\left(x\right)=n\frac{\theta }{\lambda }{\left(1+\frac{x}{\lambda }\right)}^{-\left(\theta +1\right)}\alpha^{{\left(1+\frac{x}{\lambda }\right)}^{-}\theta }[1-\text{log}\;\alpha \left(1-{\left(1+\frac{x}{\lambda }\right)}^{-\theta }\right)]\\ .{\left(\frac{\alpha \left(1-{\left(1+\frac{x}{\lambda }\right)}^{-\theta }\right)}{\alpha^{\left(1-{\left(1+\frac{x}{\lambda }\right)}^{-\theta }\right)}}\right)}^{n-1}. \end{array}$$ (12)

### 3.4 Mean Residual Life (MRL)

The MRL of GAPL is given as:

$$\begin{array}{c} MRL_{GAPL}\left(x\right)=\frac{1}{S\left(x;\left(\theta ,\alpha ,\lambda \right)\right)}\text{log}\int_{x}^{\infty }f\left(t;\left(\theta ,\alpha ,\lambda \right)\right)dt-x. \end{array}$$ (13)

where

$$\begin{array}{c} S_{GAPL}\left(x\right)=1-\frac{\alpha \left(1-{\left(1+\frac{x}{\lambda }\right)}^{-\theta }\right)}{\alpha^{\left(1-{\left(1+\frac{x}{\lambda }\right)}^{-\theta }\right)}}, \end{array}$$

and

$$\begin{array}{c} f_{GAPL}\left(x\right)=\frac{\theta }{\lambda }{\left(1+\frac{x}{\lambda }\right)}^{-\left(\theta +1\right)}\alpha^{{\left(1+\frac{x}{\lambda }\right)}^{-}\theta }[1-\text{log}\;\alpha \left(1-{\left(1+\frac{x}{\lambda }\right)}^{-\theta }\right)], \end{array}$$

$$\begin{array}{c} MRL_{GAPL}\left(x\right)=\\ \frac{\alpha^{\left(1-{\left(1+\frac{x}{\lambda }\right)}^{-\theta }\right)}}{\alpha^{\left(1-{\left(1+\frac{x}{\lambda }\right)}^{-\theta }\right)-\alpha \left(1-{\left(1+\frac{x}{\lambda }\right)}^{-\theta }\right)}}\;\text{log}\;\int_{0}^{\infty }[\frac{\theta }{\lambda }{\left(1+\frac{x}{\lambda }\right)}^{-\left(\theta +1\right)}\alpha^{{\left(1+\frac{x}{\lambda }\right)}^{-}\theta }[1-\text{log}\;\alpha \left(1-{\left(1+\frac{x}{\lambda }\right)}^{-\theta }\right)]{]}^{p}dx. \end{array}$$ (14)

### 3.5 Renyi entropy

The Renyi entropy of the GAPL distribution is given as:

$$\begin{array}{c} R_{H}\left(x\right)=\frac{1}{1-p}\;\text{log}\;\int_{0}^{\infty }f^{p}\left(x\right)dx. \end{array}$$ (15)

From Eq (6), the Renyi entropy *R*_*H*_(*x*) becomes as

$$\begin{array}{c} R_{H}\left(x\right)=\\ \frac{1}{1-p}\text{log}\int_{0}^{\infty }[\frac{\theta }{\lambda }{\left(1+\frac{x}{\lambda }\right)}^{-\left(\theta +1\right)}\alpha^{{\left(1+\frac{x}{\lambda }\right)}^{-}\theta }[1-\text{log}\;\alpha \left(1-{\left(1+\frac{x}{\lambda }\right)}^{-\theta }\right)]{]}^{p}dx. \end{array}$$ (16)

### 3.6 Skewness and kurtosis

The Moors Kurtosis and the Galton Skewness of the GAPL distribution are defined as:

$$\begin{array}{c} Z_{K}=\frac{Q\left(\frac{1}{8}\right)+Q\left(\frac{3}{4}\right)-Q\left(\frac{1}{4}\right)-Q\left(\frac{2}{4}\right)}{Q\left(\frac{3}{4}\right)-Q\left(\frac{1}{4}\right)}, \end{array}$$ (17)

and

$$\begin{array}{c} Z_{M}\left(x\right)=\frac{Q\left(\frac{1}{8}\right)+Q\left(\frac{3}{8}\right)-Q\left(\frac{5}{8}\right)-Q\left(\frac{1}{8}\right)}{Q\left(\frac{3}{4}\right)-Q\left(\frac{1}{4}\right)}. \end{array}$$ (18)

Table 2 gives the values of the Skewness (Sk) and Kurtosis (Kt).

**Table 2:** Min, Q1, Q2, Q3, Mean, Max, Std, SK, and KT.

*θ*	λ	*α*	Min	Q1	Q2	Q3	Mean	Max	Std	Sk	Kt
0.5	0.4	0.5	0.00080	0.6519	2.7068	14.9766	110.7127	10329.401	648.1126	0.7131	4.1991
		1	0.00040	0.3111	1.2000	6.0000	39.5664	3627.7179	228.1671	0.6875	3.9118
		1.5	0.00027	0.1938	0.6768	2.8850	14.9225	1306.6448	82.7292	0.6410	3.3897
		2	0.00020	0.1377	0.4399	1.5617	4.9843	375.3762	24.3857	0.5755	2.6508
		3	0.00013	0.0854	0.2407	0.6379	0.5637	6.6021	0.8989	0.4378	1.4180
	1.4	0.5	0.00280	2.2815	9.4740	52.4183	387.4945	36152.905	2268.3940	0.7131	4.1991
		1	0.00140	1.0889	4.2000	21.0000	138.4826	12697.0127	798.5849	0.6875	3.9118
		1.5	0.00093	0.6782	2.3689	10.0974	52.2287	4573.2570	289.5522	0.6410	3.3897
		2	0.00070	0.4819	1.5398	5.4658	17.4450	1313.8168	85.3500	0.5755	2.6508
		3	0.00047	0.2989	0.8426	2.2326	1.9730	23.1075	3.1460	0.4376	1.4180
	3	0.5	0.00601	4.8889	20.3014	112.3249	830.3453	77470.511	4860.8442	0.7131	4.1991
		1	0.00300	2.3333	9.0000	45.0000	296.7484	27207.884	1711.2534	0.6875	3.9118
		1.5	0.00200	1.4533	5.0762	21.6374	111.9186	9799.8364	620.4690	0.6410	3.3897
		2	0.00150	1.0328	3.2995	11.7125	37.3822	2815.3217	182.8929	0.5755	2.6508
		3	0.00100	0.6405	1.8056	4.7841	4.2278	49.5162	6.7414	0.4376	1.4180
1.5	0.4	0.5	0.00027	0.1521	0.3922	0.9500	0.8424	11.4233	1.3445	0.3983	1.2900
		1	0.00013	0.0846	0.2350	0.6079	0.5510	7.9419	0.9283	0.4253	1.3773
		1.5	0.00009	0.0563	0.1564	0.4070	0.3739	5.5357	0.6413	0.4289	1.4021
		2	0.00007	0.0415	0.1122	0.2796	0.2494	3.5176	0.4089	0.4057	1.3068
		3	0.00006	0.0267	0.0680	0.1497	0.1075	0.6386	0.1162	0.3279	0.9128
	1.4	0.5	0.00093	0.5324	1.3726	3.3249	2.9483	39.9814	4.7059	0.3983	1.2900
		1	0.00047	0.2960	0.8224	2.1278	1.9283	27.7968	3.2491	0.4253	1.3773
		1.5	0.00031	0.1971	0.5476	1.4245	1.3087	19.3749	2.2446	0.4289	1.4020
		2	0.00023	0.1451	0.3928	0.9785	0.8728	12.3115	1.4311	0.4057	1.3070
		3	0.00016	0.0933	0.2381	0.5238	0.3764	2.2351	0.4068	0.3275	0.9130
	3	0.5	0.00200	1.1408	2.9412	7.1248	6.3177	85.6745	10.0841	0.3983	1.2900
		1	0.00100	0.6342	1.7622	4.5595	4.1322	59.5646	6.9623	0.4253	1.3773
		1.5	0.00067	0.4222	1.1733	3.0526	2.8044	41.5176	4.8099	0.4289	1.4019
		2	0.00050	0.3109	0.8416	2.0969	1.8703	26.3818	3.0666	0.4057	1.3069
		3	0.00033	0.1999	0.5102	1.1224	0.8065	4.7895	0.8718	0.3273	0.9130
3	0.4	0.5	0.00013	0.0699	0.1629	0.3348	0.2500	1.7747	0.2730	0.2981	0.8717
		1	0.00007	0.0403	0.1040	0.2350	0.1779	1.4267	0.2155	0.3456	1.0050
		1.5	0.00006	0.0272	0.0718	0.1682	0.1306	1.1409	0.1676	0.3678	1.0889
		2	0.00006	0.0202	0.0526	0.1214	0.0948	0.8518	0.1221	0.3590	1.0711
		3	0.00000	0.0131	0.0326	0.0689	0.0480	0.2446	0.0478	0.2992	0.8049
	1.4	0.5	0.00047	0.2448	0.5702	1.1720	0.8749	6.2114	0.9556	0.2981	0.8717
		1	0.00023	0.1409	0.3639	0.8223	0.6227	4.9934	0.7542	0.3456	1.0050
		1.5	0.00016	0.0953	0.2512	0.5886	0.4570	3.9930	0.5866	0.3676	1.0891
		2	0.00012	0.0708	0.1843	0.4248	0.3319	2.9813	0.4274	0.3589	1.0713
		3	0.00008	0.0459	0.1144	0.2411	0.1681	0.8559	0.1672	0.2987	0.8055
	3	0.5	0.00100	0.5246	1.2218	2.5113	1.8748	13.3102	2.0478	0.2981	0.8717
		1	0.00050	0.3019	0.7798	1.7622	1.3345	10.7001	1.6160	0.3456	1.0050
		1.5	0.00033	0.2042	0.5384	1.2612	0.9793	8.5565	1.2569	0.3677	1.0891
		2	0.00025	0.1516	0.3948	0.9103	0.7112	6.3886	0.9159	0.3589	1.0714
		3	0.00017	0.0983	0.2451	0.5167	0.3602	1.8341	0.3583	0.2985	0.8053

## 4 Parameter estimation

In this section, estimation methods have been obtained for the parameters of the GAPL distribution.

### 4.1 Maximum likelihood estimation

Because the probability model’s parameters are unknown, they must be estimated using data gathered from a sample. For a more in-depth look into maximum likelihood estimate, see here [45–47]. The conventional method of maximum likelihood estimates is utilized to determine the parameter estimates in this section. The Likelihood function of the GAPL distribution is given as:

$$\begin{array}{c} L\left(\alpha ,\theta ,\lambda ;data\right)=\prod_{i=1}^{n}\;f\left(x_{i};\alpha ,\theta ,\lambda \right), \end{array}$$ (19)

Substituting from (6) in the Eq (19) expression, we get

$$\begin{array}{c} L\left(\alpha ,\theta ,\lambda ;x\right)=\frac{\theta^{n}}{\lambda^{n}}\alpha^{\sum_{i=1}^{n}{\left(1+\frac{x_{i}}{\lambda }\right)}^{-}\theta }\prod_{i=1}^{n}{\left(1+\frac{x_{i}}{\lambda }\right)}^{-\left(\theta +1\right)}[1-\text{log}\;\alpha \left(1-{\left(1+\frac{x_{i}}{\lambda }\right)}^{-\theta }\right)], \end{array}$$ (20)

by taking the log function on both sides, we get

$$\begin{array}{ccc} \ell \left(\alpha ,\theta ,\lambda ;data\right) & = & n\;\text{log}\;\theta -n\;\text{log}\;\lambda +\text{log}\;\left(\alpha \right)\sum_{i=1}^{n}{\left(1+\frac{x_{i}}{\lambda }\right)}^{-\theta }-\left(\theta +1\right)\sum_{i=1}^{n}\;\text{log}\left(1+\frac{x_{i}}{\lambda }\right)\\ & + & \sum_{i=1}^{n}[1-\left(1-{\left(1+\frac{x_{i}}{\lambda }\right)}^{-\theta }\right)\;\text{log}\;\left(\alpha \right)] \end{array}$$

Define the binomial expansion (1+xiλ)-θ=∑j=0∞(-1)j(θ+j-1j)(xiλ)j then

ℓ=nlogθ-nlogλ+log(α)∑i=1n∑j=0∞(-1)j(θ+j-1j)(xiλ)j-(θ+1)∑i=1nlog(1+xiλ)+∑i=1nlog{1-(1-∑j=0∞(-1)j(θ+j-1j)(xiλ)j)log(α)} (21)

To obtain the estimates of the parameters, the partial derivatives with respect to *α*, λ, *θ* are obtained and the results equated to zero.

∂ℓ∂α=1α∑i=1n∑j=0∞(-1)j(θ+j-1j)(xiλ)j-1α∑i=1n(1-∑j=0∞(-1)j(θ+j-1j)(xiλ)j)1-(1-∑j=0∞(-1)j(θ+j-1j)(xiλ)j)log(α), (22)

∂ℓ∂θ=nθ+∑i=1n∑j=0∞(-1)j{(θ-1)!(j-1)!-(θ+j-1)!(-1)!}(xiλ)jlog(α)j!((θ-1)!)2-∑i=1nlog(1+xiλ)+∑i=1n{(θ-1)!(j-1)!-(θ+j-1)!(-1)!j!((θ-1)!)2}(xiλ)jlog(α)1-(1-∑j=0∞(-1)j(θ+j-1j)(xiλ)j)log(α), (23)

and

∂ℓ∂λ=-nλ-log(α)∑i=1n∑j=0∞j!(-1)jxij(θ+j-1j)(1λ)j-1+(θ+1)∑i=1n(xiλ2+λxi)-∑i=1n∑j=0∞j!(-1)jxij(θ+j-1j)(1λ)2jlog(α)1-(1-∑j=0∞(-1)j(θ+j-1j)(xiλ)j)log(α) (24)

Eqs (22)–(24) are not in closed form. To obtain the solution, numerical methods are proposed.

### 4.2 Bootstrap confidence intervals

The last section demonstrated how difficult it is to derive second-order derivatives in order to generate ACIs for the unknown model parameters. So, we take bootstrapping into account. In particular, we use the percentile bootstrap (Boot-p) and bootstrap-t (Boot-t) approaches (Tibshirani [48]) and bootstrap-t (Boot-t) (see Hall [49]) respectively.

### 4.3 Parametric Boot-p CI

Here, we’ll go over the formula for getting confidence intervals using the Boot-p approach. Initially, we get the MLEs of Θ = (*α*, λ, *θ*), by solving Eq 20. Also, denoted them by $\hat{\Theta }=\left(\hat{\alpha },\hat{\lambda },\hat{\theta }\right)$ then, the bootstrap sample $x^{*}=\left(x_{1}^{*},x_{2}^{*},\cdots ,x_{m}^{*}\right)$ has to be generated. We compute ${\hat{\Theta }}^{*}=\left({\hat{\lambda }}^{*},{\hat{\alpha }}^{*},{\hat{\theta }}^{*}\right)$ based on x*. Repeat this procedure for *Nboot* times to get ${\hat{\Theta }}_{1}^{*},{\hat{\Theta }}_{2}^{*},\cdots ,{\hat{\Theta }}_{Nboot}^{*}$ where ${\hat{\Theta }}_{i}^{*}=\left({\hat{\alpha_{j}}}_{i}^{*},{\hat{\lambda_{j}}}_{i}^{*},{\hat{\theta_{j}}}_{i}^{*}\right)$, *i* = 1, 2, ⋯, *Nboot*. Next, we arrange ${\hat{\Theta }}_{i}^{*}$ in ascending order and denote them by ${\hat{\Theta }}_{\left(1\right)}^{*},{\hat{\Theta }}_{\left(2\right)}^{*},\cdots ,{\hat{\Theta }}_{\left(Nboot\right)}^{*}$. Thus, the (1 − *δ*)100% approximate bootstrap-p confidence interval for Θ is obtained as (*L*, *U*), where $L={\hat{\Theta }}_{Nboot\left(\frac{\delta }{2}\right)}^{*}$ and $U={\hat{\Theta }}_{Nboot\left(1-\frac{\delta }{2}\right)}^{*}$.

### 4.4 Parametric Boot-t CI

Under a small sample size, the Boot-p approach does not perform well; for further information. Because the Boot-t approach is easier to use than the Boot-p method, we will examine it in this subsection. We get ${\hat{\Theta }}^{*}=\left({\hat{\alpha }}^{*},{\hat{\lambda }}^{*},{\hat{\theta }}^{*}\right)$, similar to the procedure as mentioned in Boot-p method. Then, based on the bootstrap sample $x^{*}=x_{1}^{*},x_{2}^{*},\cdots ,x_{m}^{*}$, we compute the variance-covariance matrix ${\hat{I}}_{x^{*}}^{-1}\left({\hat{\alpha_{j}}}^{*},{\hat{\lambda_{j}}}^{*},{\hat{\theta_{j}}}^{*}\right)$. For *i* = 1, 2, ⋯, *Nboot*, calculate the value of the statistic $T_{\Theta_{i}}^{*}=\frac{{\hat{\Theta }}_{i}^{*}-{\hat{\Theta }}_{i}}{\sqrt{\hat{var\left(\Theta_{i}^{*}\right)}}}$. Then, we arrange them in ascending order and get $T_{\Theta_{\left(1\right)}}^{*},T_{\Theta_{\left(2\right)}}^{*},\cdots ,T_{\Theta_{\left(Nboot\right)}}^{*}$. Thus, the (1 − *δ*)100% approximate bootstrap-t confidence interval for Θ is obtained as (*L*, *U*), where $L={\hat{\Theta }}_{i}+\sqrt{\hat{var\left(\Theta_{i}^{*}\right)}}*T_{\Theta_{\left(Nboot\left(\frac{\delta }{2}\right)\right)}}^{*}$ and $U={\hat{\Theta }}_{i}+\sqrt{\hat{var\left(\Theta_{i}^{*}\right)}}*T_{\Theta_{\left(Nboot\left(1-\frac{\delta }{2}\right)\right)}}^{*}$.

## 5 Bayesian estimation method

In this section, Bayesian inference was used to estimate the GAPL distribution parameters using an informative prior in order to achieve the correct posterior distributions. For more information and examples of the Bayesian estimation method, see [23, 50–55].

### 5.1 The model parameter priors

In informative priors, it’s noteworthy to notice that when the three GAPL distribution parameters are unknown, a joint conjugate prior to the parameters does not exist. As a result, we investigate Bayesian inference using independent gamma priors for d and q, as well as the subsequent combined prior distribution:

$$\begin{array}{c} \pi \left(\alpha ,\lambda ,\theta \right)\propto \alpha^{q_{1}-1}\lambda^{q_{2}-1}\theta^{q_{3}-1}e^{-\left(w_{1}\alpha +w_{2}\lambda +w_{3}\theta \right)},\;\alpha ,\lambda ,\theta >0, \end{array}$$ (25)

The hyper-parameters *q*_*i*_, *w*_*i*_, *i* = 1, 2, 3 are chosen to reflect prior information about the parameters of the GAPL distribution *α*, λ and *θ*, and they should be well-known and positive.

### 5.2 Posterior distribution

The likelihood function Eq (26) as follows:

$$\begin{array}{c} L\left(\alpha ,\theta ,\lambda ;data\right)=\alpha^{\sum_{i=1}^{n}{\left(1+\frac{x_{i}}{\lambda }\right)}^{-}\theta }\prod_{i=1}^{n}{\left(1+\frac{x_{i}}{\lambda }\right)}^{-\left(\theta +1\right)}[1-\text{log}\;\alpha \left(1-{\left(1+\frac{x_{i}}{\lambda }\right)}^{-\theta }\right)], \end{array}$$ (26)

and the joint prior function Eq (25) can express the joint posterior distribution. Consequently, Θ joint posterior density function is

$$\begin{array}{c} \begin{array}{cc} \Pi \left(\alpha ,\theta ,\lambda ;data\right)= & B\;\alpha^{q_{1}-1}\lambda^{q_{2}-1}\theta^{q_{3}-1}e^{-\left(w_{1}\alpha +w_{2}\lambda +w_{3}\theta \right)}\alpha^{\sum_{i=1}^{n}{\left(1+\frac{x_{i}}{\lambda }\right)}^{-\theta }}\prod_{i=1}^{n}{\left(1+\frac{x_{i}}{\lambda }\right)}^{-\left(\theta +1\right)}\\ & \times [1-\text{log}\;\alpha \left(1-{\left(1+\frac{x_{i}}{\lambda }\right)}^{-\theta }\right)]. \end{array} \end{array}$$ (27)

The posterior density normalization constant B, which in practice frequently requires an integral over the parameter space, is typically intractable as follows:

$$\;B=\int_{0}^{\infty }\int_{0}^{\infty }\int_{0}^{\infty }\alpha^{q_{1}-1}\lambda^{q_{2}-1}\theta^{q_{3}-1}e^{-\left(w_{1}\alpha +w_{2}\lambda +w_{3}\theta \right)}\alpha^{\sum_{i=1}^{n}{\left(1+\frac{x_{i}}{\lambda }\right)}^{-}\theta }$$

$$\times \prod_{i=1}^{n}{\left(1+\frac{x_{i}}{\lambda }\right)}^{-\left(\theta +1\right)}[1-\text{log}\;\alpha \left(1-{\left(1+\frac{x_{i}}{\lambda }\right)}^{-\theta }\right)]d\alpha d\lambda d\theta .$$

### 5.3 Loss functions

The squared-error loss function, which is denoted by SELF, is the symmetric loss function. The average is then the Bayesian estimator of Θ under SELF.

$$\begin{array}{c} \tilde{\Theta }=E_{\Theta }\left(\Theta \right). \end{array}$$ (28)

The two most well-known asymmetric loss functions are the LINEX and the entropy loss functions. Varian [56] introduced an extremely helpful asymmetric loss function, which has recently been used in several publications by [52, 57, 58].

The shape of this loss function, where *c* ≠ 0, depends on the value of *c*. When the LINEX loss function is used, the Bayes estimator of Θ is

$$\begin{array}{c} \tilde{\Theta }=\frac{-1}{c}\;\text{ln}\;[E_{\Theta }\left(e^{-c\;\Theta }\right)]. \end{array}$$ (29)

According to Calabria and Pulcini [59], the entropy loss function is a decent asymmetric loss function.

The entropy loss function’s Bayesian estimation for the constant Θ is

$$\begin{array}{c} \tilde{\Theta }=[E\Theta \left(\Theta^{-c}\right){]}^{\frac{-1}{c}}, \end{array}$$ (30)

As expected, the conditional distributions of *α*, λ, and *θ* cannot be analytically reduced to any standard distribution comparable to Bayesian inference, in this case, using the loss function approach. As a result, we recommend using the MCMC simulation technique to approximate the Bayesian estimates of *α*, λ, and *θ*.

### 5.4 Markov chain Monte Carlo

The MCMC method will be used since the expectation of loss functions are challenging to answer analytically by mathematical integration. The most important sub-classes of MCMC algorithms are Gibbs sampling and the more general Metropolis-within-Gibbs samplers. This algorithm was discussed in Robert et al. [60]. The Metropolis-Hastings (MH) algorithm, like acceptance-rejection sampling, treats a candidate value generated from a proposal distribution as normal for each iteration of the process. Starting at $\Theta_{i}={\hat{\Theta }}_{i}$, the MH method computes an appropriate transition in two steps:

(1) Draw *π*(Θ*|Θ) from a proposal density while Θ* is a constant.

(2) You can either stick with the current sample Θ_*i*+1_ = Θ or switch to Θ_*i*+1_ = Θ*. with acceptance likelihood.

$$\begin{array}{c} a_{\Theta^{*}|\Theta }=min[1,\frac{G\left(\Theta^{*}|x\right)\pi \left(\Theta \right)}{G\left(\Theta |x\right)\pi \left(\Theta^{*}|\Theta \right)}]. \end{array}$$

This well-stated transition density ensures that the chain converges to its particular invariant density starting from any initial condition, in addition to ensuring that the target density remains invariant.

## 6 Simulation studies and results

In this section, the simulation studies and results have been shown.

### 6.1 Monte Carlo simulation

The average bias, root mean square error, and mean of the parameter estimates were assessed through a simulation study. Different sample sizes and different sets of parameter values were used in the simulation study. The Average Bias (AB) and the Root Mean Squared Error (RMSE) were calculated using the equations below:

$$\begin{array}{c} AB=\frac{1}{N}\sum_{i=1}^{N}\left(\hat{\phi }-\phi \right), \end{array}$$ (31)

where *ϕ* is a vector of parameters (λ, *α*, *θ*) and

$$\begin{array}{c} RMSE=\sqrt{\frac{1}{N}\sum_{i=1}^{N}{\left(\hat{\phi }-\phi \right)}^{2}} \end{array}$$ (32)

**Fig 3:**
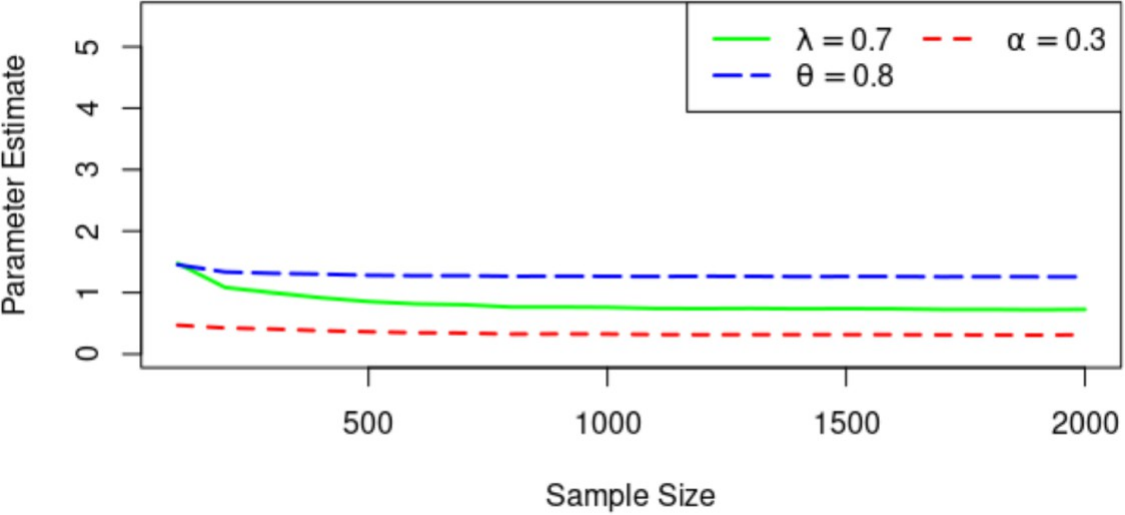
Plots of the MLEs against sample size.

**Fig 4:**
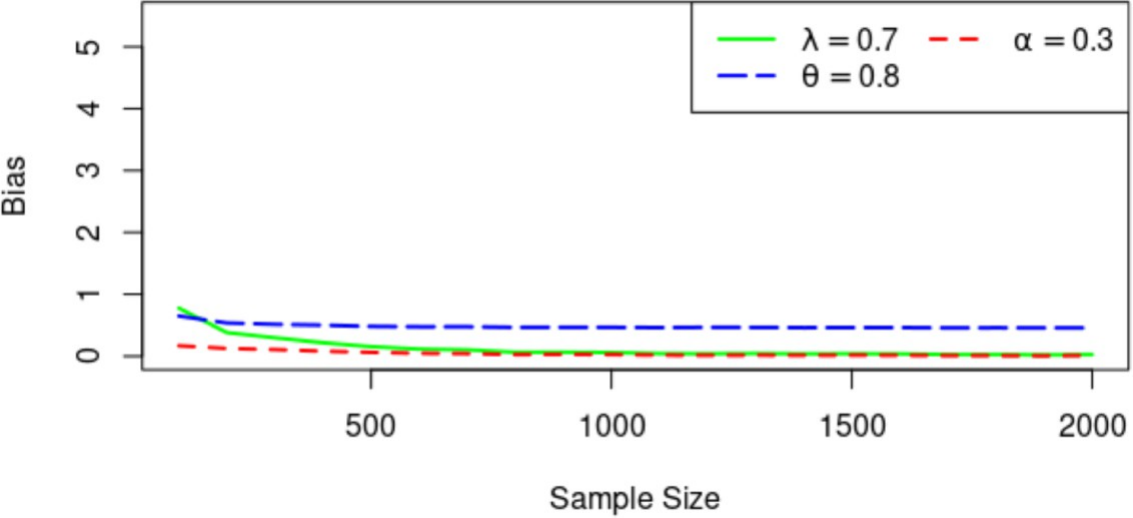
Average bias for estimators.

**Fig 5:**
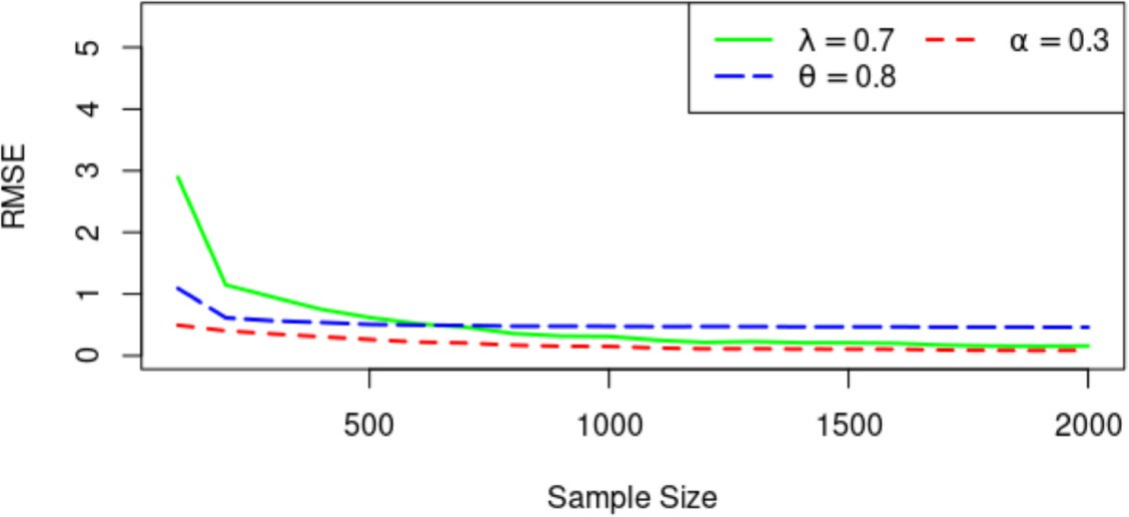
RMSE for estimators.

Figs 3–5 display the Means of the parameter estimates, the RMSE and AB for the MLEs (λ, *α*, *θ*) = (0.7, 0.3, 0.8) for increasing sample sizes. As observed, the parameter values tend to be the true value as the sample size increases. For the RMSE and AB, they decrease as the sample size increases.

Tables 3–5 discussed different cases as Case I is *α* = 1.2, *θ* = 1.5 with λ is changed as 0.7, 1.5, and 3, Case II is *α* = 1.2, *θ* = 3 with λ is changed as 0.7, 1.5, and 3, and Case III is λ = 1.5, *θ* = 0.7 with *α* is changed as 0.7, 1.5, and 3. The sample size is changed to 50, 100, and 200. These tables obtained AB, MSE, CI with length as length asymptotic CI (LACI), length credible CI (LCCI), length asymptotic CI (LACI), length bootstrap-p for MLE (LBP.MLE), length bootstrap-t for MLE (LBt.MLE), length bootstrap-p for Bayes (LBP.Bayes), and length bootstrap-t for Bayes (LBt.Bayes).

**Table 3:** AB, MSE, CI for MLE and Bayesian estimation of parameters of GAPL: Case I.

*α* = 1.2; *θ* = 1.5			MLE		Bayesian		CI		Bootstraping			
λ	n		AB	MSE	AB	MSE	LACI	LCCI	LBP.MLE	LBt.MLE	LBP.Bayes	LBt.Bayes
0.7	50	*α*	-0.0412	0.2370	0.1751	0.2233	1.9026	1.7214	0.0609	0.0613	0.0544	0.0541
		λ	0.2228	0.6933	0.1968	0.1693	3.1465	1.4173	0.1036	0.1068	0.0461	0.0461
		*θ*	0.3467	1.2927	0.0712	0.0935	4.2469	1.1664	0.1378	0.1417	0.0365	0.0374
	100	*α*	-0.0464	0.2306	0.1104	0.0961	1.8745	1.1358	0.0592	0.0597	0.0348	0.0349
		λ	0.2022	0.6650	0.1542	0.0909	3.0984	1.0166	0.0995	0.1011	0.0336	0.0331
		*θ*	0.3107	1.2033	0.0547	0.0396	4.1361	0.7502	0.1367	0.1383	0.0235	0.0232
	200	*α*	-0.0316	0.2140	0.0882	0.0534	1.7917	0.8381	0.0600	0.0593	0.0280	0.0283
		λ	0.1921	0.5442	0.1170	0.0495	2.7710	0.7423	0.0875	0.0880	0.0227	0.0227
		*θ*	0.2349	1.1620	0.0519	0.0227	4.0007	0.5541	0.1264	0.1271	0.0173	0.0173
1.5	50	*α*	-0.0625	0.3011	0.1430	0.1427	2.1387	1.3716	0.0823	0.0812	0.0528	0.0515
		λ	0.2167	1.7450	0.2289	0.1944	5.1120	1.4786	0.1985	0.2001	0.0551	0.0551
		*θ*	0.2360	0.9695	0.0635	0.0770	3.7499	1.0595	0.1428	0.1456	0.0410	0.0409
	100	*α*	-0.0475	0.1920	0.1618	0.1383	1.7083	1.2553	0.0541	0.0533	0.0491	0.0497
		λ	0.0518	0.4829	0.2042	0.1878	2.7178	1.4099	0.0852	0.0891	0.0475	0.0470
		*θ*	0.0829	0.2533	0.0130	0.0640	1.9469	0.9907	0.0618	0.0618	0.0307	0.0296
	200	*α*	-0.0318	0.1820	0.1069	0.0697	1.7698	0.9465	0.0578	0.0573	0.0301	0.0307
		λ	-0.0096	0.3709	0.1403	0.0742	2.3882	0.9154	0.0744	0.0745	0.0282	0.0285
		*θ*	0.0308	0.2026	0.0156	0.0295	1.7612	0.6710	0.0563	0.0564	0.0216	0.0216
3	50	*α*	-0.0654	0.2957	0.1344	0.1733	2.1171	1.5454	0.0663	0.0656	0.0518	0.0525
		λ	0.2263	2.7739	0.1386	0.0781	6.4715	0.9515	0.2047	0.2064	0.0293	0.0291
		*θ*	0.1944	0.5144	0.0280	0.0734	2.7077	1.0568	0.0814	0.0814	0.0326	0.0327
	100	*α*	-0.0344	0.2767	0.0744	0.0566	2.0585	0.8859	0.0662	0.0662	0.0284	0.0285
		λ	0.1519	2.4772	0.0738	0.0232	6.1440	0.5222	0.1966	0.1974	0.0166	0.0166
		*θ*	0.1564	0.4537	0.0258	0.0285	2.6581	0.6543	0.0810	0.0901	0.0202	0.0203
	200	*α*	-0.0762	0.2750	0.0441	0.0364	2.0350	0.7284	0.0659	0.0648	0.0227	0.0226
		λ	0.1355	2.1750	0.0525	0.0113	5.7596	0.3623	0.1781	0.1781	0.0115	0.0119
		*θ*	0.1410	0.4255	0.0250	0.0191	2.5672	0.5329	0.0808	0.0819	0.0168	0.0169

**Table 4:** AB, MSE, CI for MLE and Bayesian estimation of parameters of GAPL: Case II.

*α* = 1.2; *θ* = 3			MLE		Bayesian		CI		Bootstraping			
λ	n		AB	MSE	AB	MSE	LACI	LCCI	LBP.MLE	LBt.MLE	LBP.Bayes	LBt.Bayes
0.7	50	*α*	-0.0935	0.2542	0.1997	0.2374	1.9431	1.1884	0.0625	0.0626	0.0366	0.0372
		λ	0.0259	0.1997	0.1908	0.1492	1.7495	1.5459	0.0558	0.0556	0.1132	0.1379
		*θ*	0.2284	1.8611	0.0587	0.0529	5.2749	4.4760	0.1649	0.1648	1.2584	1.3382
	100	*α*	-0.1130	0.2474	0.1444	0.1258	1.8004	1.1264	0.0624	0.0624	0.0359	0.0348
		λ	0.0164	0.1602	0.1455	0.0811	1.5685	1.4938	0.0479	0.0477	0.0616	0.0661
		*θ*	0.2310	1.4931	0.0408	0.0170	4.7060	4.2041	0.1562	0.1576	0.4779	0.4780
	200	*α*	-0.0977	0.2390	0.1125	0.0796	1.4201	1.1045	0.0613	0.0618	0.0425	0.0381
		λ	0.0493	0.1523	0.1089	0.0439	1.3876	1.3789	0.0459	0.0459	0.0512	0.0613
		*θ*	0.2305	1.0890	0.0236	0.0079	4.2575	4.1516	0.1561	0.1516	0.4679	0.4180
1.5	50	*α*	-0.0129	0.3159	0.1997	0.2374	1.9431	1.7432	0.0786	0.0796	0.0560	0.0559
		λ	0.2115	1.0186	0.1908	0.1492	1.7495	1.3169	0.0558	0.0556	0.0408	0.0408
		*θ*	0.4131	2.9782	0.0587	0.0529	5.2749	0.8720	0.1649	0.1648	0.0276	0.0276
	100	*α*	-0.0622	0.2755	0.1444	0.1258	1.9004	1.2707	0.0755	0.0787	0.0419	0.0422
		λ	0.2077	0.9141	0.1455	0.0811	1.5685	0.9602	0.0588	0.0585	0.0296	0.0296
		*θ*	0.4062	2.8351	0.0408	0.0170	4.7060	0.4852	0.1607	0.1640	0.0150	0.0150
	200	*α*	-0.0379	0.2386	0.1125	0.0796	1.8942	1.0145	0.0640	0.0639	0.0319	0.0319
		λ	0.1923	0.8250	0.1089	0.0439	1.4876	0.7025	0.0479	0.0477	0.0233	0.0232
		*θ*	0.3498	1.5804	0.0236	0.0079	4.2575	0.3354	0.1562	0.1576	0.0107	0.0107
3	50	*α*	-0.0480	0.2754	0.0783	0.0783	2.0496	1.0540	0.0941	0.1038	0.0341	0.0336
		λ	0.0609	2.1712	0.1519	0.0883	5.7741	1.0018	0.1866	0.1868	0.0333	0.0332
		*θ*	0.2521	2.2707	0.0379	0.0491	5.8267	0.8558	0.1907	0.1938	0.0267	0.0266
	100	*α*	-0.0399	0.2592	0.0537	0.0466	2.0124	0.8198	0.0661	0.0661	0.0260	0.0260
		λ	0.0592	2.0352	0.0771	0.0274	5.2004	0.5747	0.1847	0.1848	0.0190	0.0189
		*θ*	0.2957	2.1578	0.0307	0.0195	5.1893	0.5345	0.1850	0.1853	0.0165	0.0166
	200	*α*	-0.0374	0.2315	0.0355	0.0343	2.0018	0.7132	0.0657	0.0370	0.0223	0.0223
		λ	0.0488	1.9230	0.0426	0.0132	5.0847	0.4185	0.1758	0.1763	0.0133	0.0133
		*θ*	0.2349	1.7908	0.0211	0.0111	4.4076	0.4043	0.1941	0.1930	0.0128	0.0128

**Table 5:** AB, MSE, CI for MLE and Bayesian estimation of parameters of GAPL: Case III.

λ = 1.5; *θ* = 0.7			MLE		Bayesian		CI		Bootstraping			
*α*	n		AB	MSE	AB	MSE	LACI	LCCI	LBP.MLE	LBt.MLE	LBP.Bayes	LBt.Bayes
0.7	50	*α*	-0.0009	0.2087	0.1826	0.1986	1.7919	1.5947	0.0632	0.0632	0.0548	0.0546
		λ	0.0020	0.5778	0.2274	0.2301	2.9815	1.6566	0.1051	0.1053	0.0580	0.0596
		*θ*	0.0212	0.0330	0.0092	0.0235	0.7077	0.6006	0.0262	0.0263	0.0201	0.0201
	100	*α*	-0.0004	0.1322	0.1552	0.1560	1.4258	1.4246	0.0436	0.0434	0.0480	0.0481
		λ	0.0314	0.3005	0.2347	0.2153	2.1463	1.5699	0.0641	0.0640	0.0497	0.0497
		*θ*	0.0154	0.0163	0.0014	0.0143	0.4965	0.4689	0.0160	0.0156	0.0151	0.0150
	200	*α*	-0.0064	0.1208	0.1362	0.1134	1.3629	1.2076	0.0416	0.0413	0.0385	0.0378
		λ	0.0324	0.2690	0.1666	0.0892	2.0303	0.9722	0.0652	0.0649	0.0299	0.0298
		*θ*	0.0195	0.0155	0.0009	0.0119	0.4823	0.4283	0.0152	0.0152	0.0137	0.0139
1.5	50	*α*	-0.0692	0.2244	0.4172	0.2072	1.8683	1.6938	0.3374	0.3272	0.5403	0.5511
		λ	0.1346	0.5348	0.1783	0.2151	2.8658	1.7071	0.4978	0.5112	0.3092	0.3180
		*θ*	0.0868	0.0928	-0.0254	0.0409	1.1639	0.7999	0.2063	0.2176	0.1461	0.1454
	100	*α*	-0.0895	0.2177	0.2529	0.1959	2.0351	2.8414	0.0689	0.0699	0.0888	0.0907
		λ	-0.0631	0.3060	0.0798	0.1294	2.1553	1.3755	0.0704	0.0704	0.0423	0.0423
		*θ*	0.0246	0.0359	-0.0123	0.0313	0.7373	0.6926	0.0227	0.0226	0.0218	0.0217
	200	*α*	-0.0799	0.1927	0.0967	0.1304	2.0058	1.3647	0.0619	0.0618	0.0442	0.0450
		λ	-0.0569	0.2320	0.0719	0.0561	2.2056	0.8854	0.0711	0.0708	0.0279	0.0280
		*θ*	0.0213	0.0332	0.0098	0.0157	0.7098	0.4897	0.0220	0.0218	0.0156	0.0157
3	50	*α*	-0.0299	2.1473	0.2006	0.7729	5.7459	3.3569	0.1862	0.1862	0.1052	0.1109
		λ	0.1158	0.7939	0.1093	0.1592	3.4649	1.5050	0.1117	0.1117	0.0489	0.0493
		*θ*	0.1316	0.1242	0.0582	0.0602	1.2822	0.9344	0.0403	0.0396	0.0295	0.0291
	100	*α*	0.0388	2.0266	0.0685	0.1505	5.0866	1.4974	0.1722	0.1712	0.0437	0.0448
		λ	0.1455	0.7826	0.1050	0.0634	3.4224	0.8976	0.1084	0.1089	0.0285	0.0285
		*θ*	0.1391	0.1201	0.0498	0.0250	1.2305	0.5890	0.0402	0.0401	0.0193	0.0192
	200	*α*	0.0188	1.0571	0.0104	0.0346	4.6463	0.7287	0.1531	0.1521	0.0238	0.0237
		λ	0.1268	0.6866	0.0735	0.0369	3.2615	0.6963	0.1016	0.1013	0.0216	0.0217
		*θ*	0.1262	0.1200	0.0482	0.0182	1.1283	0.4940	0.0401	0.0401	0.0158	0.0159

#### 6.1.1 Remarks on simulation

1. The values of the parameter estimates approach the true value as the sample size increases.
2. The RMSE of the parameters decreases with an increase in sample size.
3. The AB of the parameter estimates decreases with an increase in sample size.

## 7 Applications to COVID-19 data set

The data will be applied to illustrate the flexibility and importance of the GAPL distribution with its sub-model (Lomax distribution) and other competing model (power Lomax) and Generalized exponential distributions. The estimation of the unknown parameters will be obtained by the ML method. The values of the models of the statistics log-likelihood, Akaike Information Criterion(AIC), Bayesian Information Criterion (BIC), and Consistent Information Criterion (CAIC) are used to compare the candidate distributions. In general the smaller the values the appropriate the distributions to fit the data. The Mathematical formulas of the criterion are:

$$\begin{array}{c} AIC=-2L\left(\hat{\phi }\right)+2h \end{array}$$

$$\begin{array}{c} BIC-2L\left(\hat{\phi }\right)+h\;\text{log}\;n \end{array}$$

$$\begin{array}{c} HQIC=-2L\left(\hat{\phi }\right)+2h\;\text{log}\left(\text{log}\;n\right) \end{array}$$

$$\begin{array}{c} CAIC=-2L\left(\hat{\phi }\right)+\frac{2hn}{n-h-1} \end{array}$$

where $L\left(\hat{\phi }\right)$ denotes the log-likelihood function evaluated at the maximum likelihood estimation, *h* is the number of parameters and *n* is the sample size. Here we let *ϕ* denote (λ, *θ*, *α*).

The proposed distribution is compared to the following distributions:

1. Exponential Lomax distribution [61] with CDF given as
  $$F\left(x\right)=1-e^{-\lambda {\left(\frac{\beta }{x+\beta }\right)}^{-\alpha }}$$
2. Lomax distribution [27] with CDF
  $$F\left(x\right)=1-{\left(\frac{\beta }{x+\beta }\right)}^{\alpha }$$
3. The Power Lomax [62] with CDF given as
  $$F\left(x\right)=1-\lambda^{\alpha }{\left(x^{\beta }+\lambda \right)}^{-\alpha }$$

### 7.1 Data set I: China COVID-19 survival times

The survival rates of patients affected by the COVID-19 pandemic in China are discussed in this subsection. The data set under consideration shows how long patients lived after being admitted to the hospital until they passed away. A group of fifty-three (53) COVID-19 sufferers were among them. From January to February 2020, they were discovered in hospitals in critical condition [63]. The descriptive statistics for the data are displayed in Table 6. The data is right skewed because of the positive sign of the skewness coefficient.

**Table 6:** Summary statistics for China COVID-19 daily cases data.

Statistic	Min	Max	Mean	Std.dev	Median	Kurtosis	Skewness
Value	3.000	150.00	49.742	43.873	33.000	-0.623	0.817

**Fig 6:**
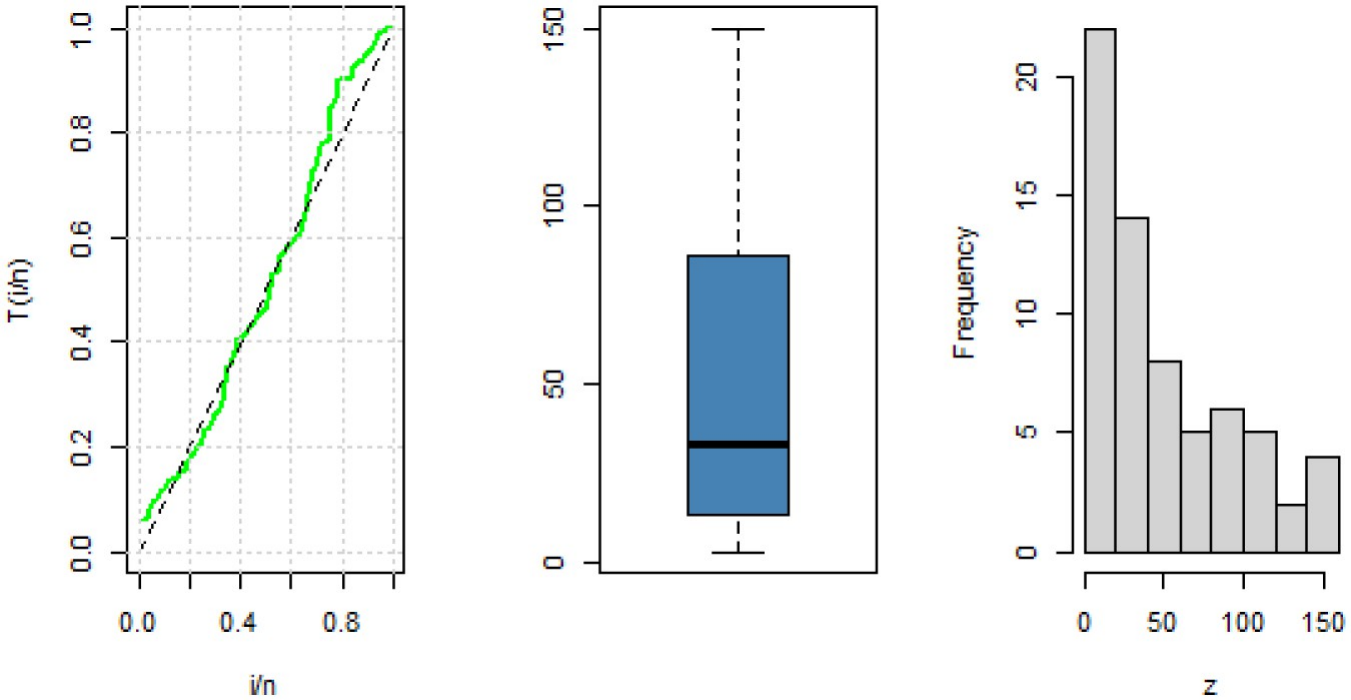
TTT-transform plot, boxplot and histogram for China COVID-19 daily number of cases.

The data has a modified bathtub failure rate as depicted in the TTT plot in Fig 6.

The MLEs of the parameters of the proposed distribution GAPL and its sub-models are presented in Table 7.

**Table 7:** MLEs and SE (in parenthesis) for China COVID-19 survival data.

Model	$\hat{\lambda }$	$\hat{\theta }$	$\hat{\alpha }$
GAPL	16.205(14.852)	1.657(0.530)	0.062 (0.110)
Power Lomax	1.105(0.401)	28.956(8.479)	1.025(0.136)
Lomax	25.816(7.356)	1.107(0.232)	-

The GAPL distribution provides a better fit than the competing distributions. As indicated in Table 8, the GAPL distribution has the highest log-likelihood and the smallest values of K-S and W* compared to the other models. Considering the formal tests of goodness of fit tests, in order to verify which distributions better fit the china daily COVID-19 cases data, since the GAPL distribution has the lowest values for the K-S, Anderson-Darling, and W* we then conclude that the distribution provides a better fit than the competing distributions.

**Table 8:** Log-likelihood, information criteria and goodness-of-fit statistics for China daily COVID-19 cases data.

Model	*l*	AIC	A*	K-S	p-value	W*	BIC	CAIC
GAPL	-327.902	661.804	1.118	0.096	0.565	0.155	668.32	662.110
Lomax	-335.099	674.199	1.021	0.161	0.064	0.142	678.578	674.389
Power Lomax	-334.126	674.252	1.026	0.151	0.097	0.143	680.82	674.601

**Fig 7:**
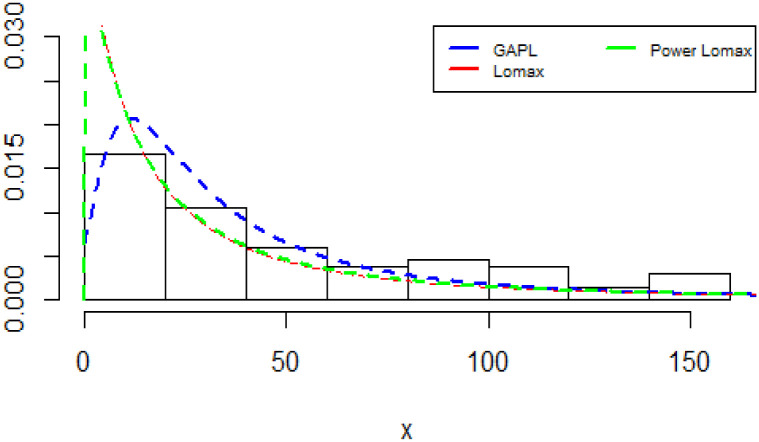
Fitted Densities plot for China COVID-19 daily cases.

The plots of the densities of the fitted distributions are shown in Fig 7.

**Fig 8:**
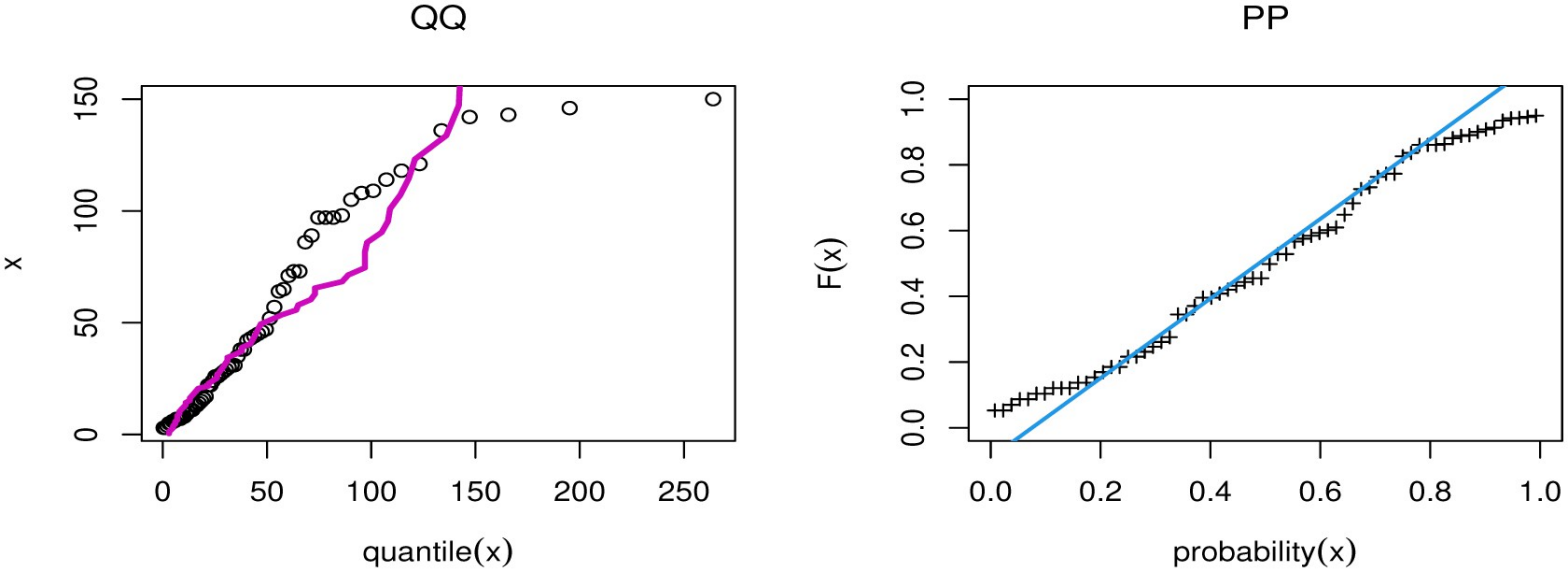
PP and QQ plots of GAPL distribution for China COVID-19 daily cases.

**Fig 9:**
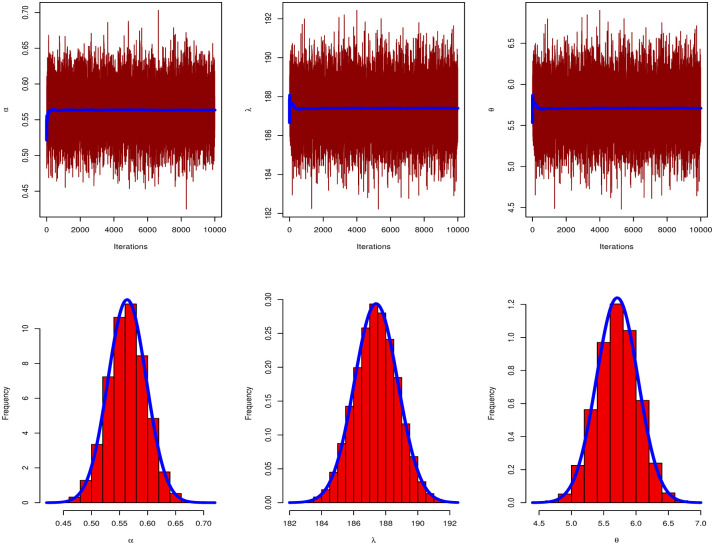
MCMC plot of GAPL distribution for China COVID-19 daily cases.

Table 9 discussed Bayesian estimation for parameters of GAPL for China COVID-19 daily cases. By comparing Bayesian and MLE in Table 7, we note that the Bayesian estimation has the smallest SE for parameters. Fig 8 presents the PP and QQ plots of GAPL distribution for China’s COVID-19 daily cases. Fig 9 shows MCMC plots of GAPL parameters for China COVID-19 daily cases.

**Table 9:** Bayesian estimation for parameters of GAPL for China COVID-19 daily cases.

	*α*	λ	*θ*
estimates	0.5635	187.3994	5.7087
SE	0.0342	1.3567	0.3216

### 7.2 Data set II: Netherlands COVID-19 mortality rates

In this subsection, the data set under consideration shows how long patients lived after being admitted to the hospital until they passed away. This data is available at this link (https://ourworldindata.org/coronavirus/country/netherlands).

The descriptive statistics for the data are displayed in Table 10. The data is right skewed because of the positive sign of the skewness coefficient.

**Table 10:** Summary statistics for China COVID-19 daily cases data.

Statistic	Min	Max	Mean	Std.dev	Median	Kurtosis	Skewness
Value	1.273	14.918	16.156	3.533	5.369	-0.240	0.792

**Fig 10:**
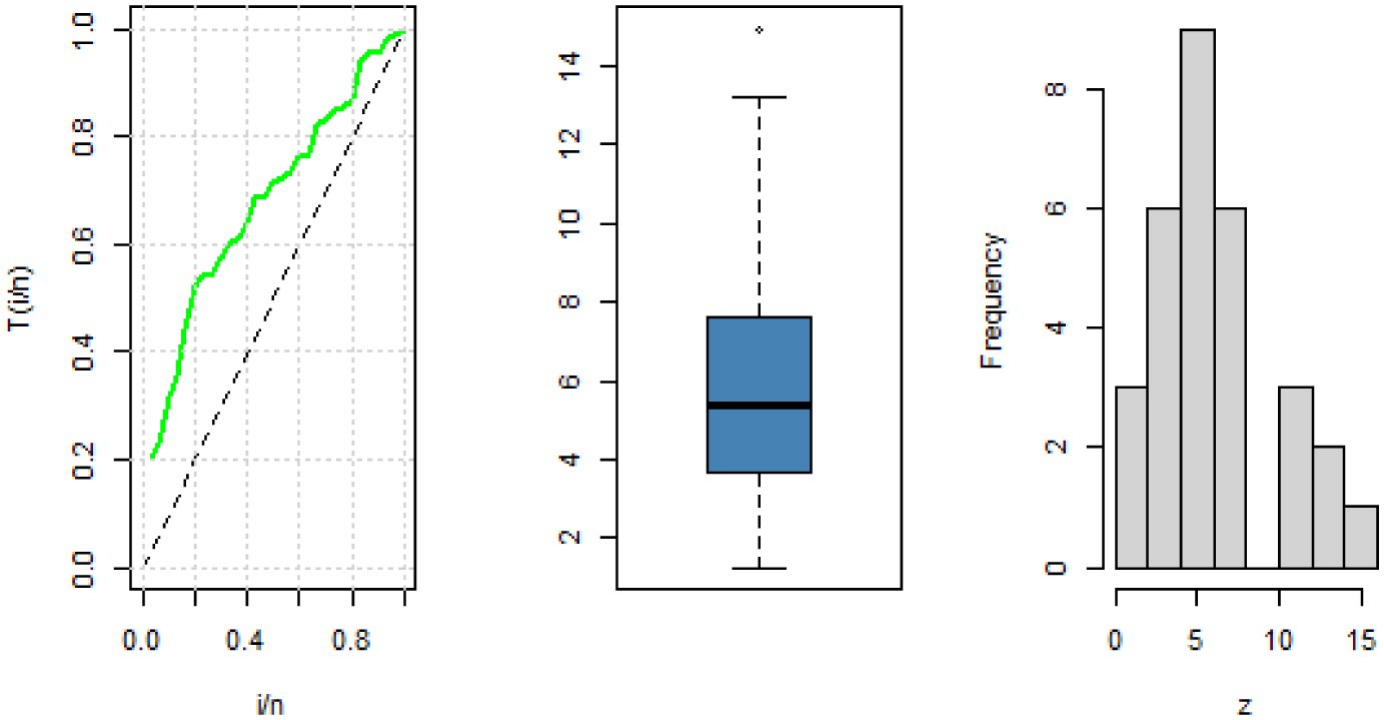
TTT-transform plot, boxplot, and histogram for Netherlands COVID-19 Mortality rates.

The data has an increasing failure rate as depicted in the TTT plot in Fig 10.

The maximum likelihood estimates of the parameters of the proposed distribution GAPL and its sub-models are presented in Table 11.

**Table 11:** MLEs and SE (in parenthesis) for China COVID-19 survival data.

Model	$\hat{\lambda }$	$\hat{\theta }$	$\hat{\alpha }$
GAPL	10.586(9.28)	5.51(3.41)	0.0034 (0.006)
Power Lomax	0.792(0.417)	13.496(5.408)	2.029(0.466)
Exponential Lomax	0.005(0.004)	0.547(0.394)	1.986(0.364)
Lomax	18.97(9.09)	3.68(1.648)	-

**Fig 11:**
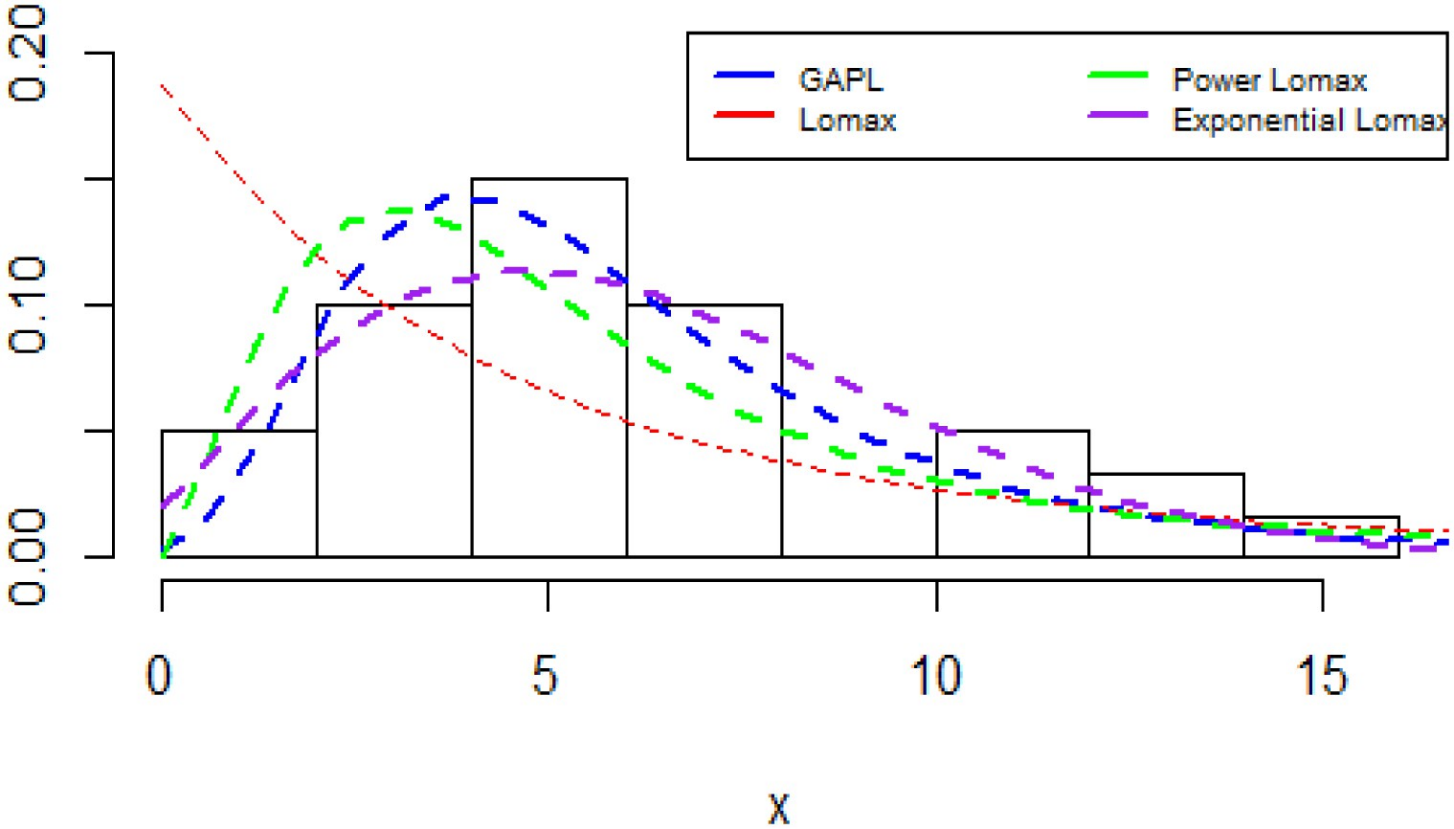
Fitted Densities plot for Netherlands COVID-19 mortality dates data.

The GAPL distribution provides a better fit than its competing distributions. As indicated in Table 12, the GAPL distribution has the highest log-likelihood and the smallest values of K-S and W* compared to the other models. Considering the formal tests of goodness of fit tests, in order to verify which distributions better fit the jet airplane data, since the GAPL distribution has the lowest values for the K-S, Anderson-Darling, and W* we then conclude that the distribution provides a better fit than the sub-models. The plots of the densities of the fitted distributions are shown in Fig 11.

**Table 12:** Log-likelihood, information criteria and goodness-of-fit statistics for China daily COVID-19 cases data.

Model	*l*	AIC	A*	K-S	p-value	W*	BIC	CAIC
GAPL	-77.31	160.61	0.186	0.0734	0.9931	0.0212	164.82	161.54
Lomax	-87.39	178.77	0.179	0.293	0.008	0.022	181.57	179.22
Exponential Lomax	-77.55	161.12	0.357	0.096	0.9185	0.058	165.32	162.04
Power Lomax	-82.19	170.38	0.322	0.237	0.0573	0.039	174.58	171.30

**Fig 12:**
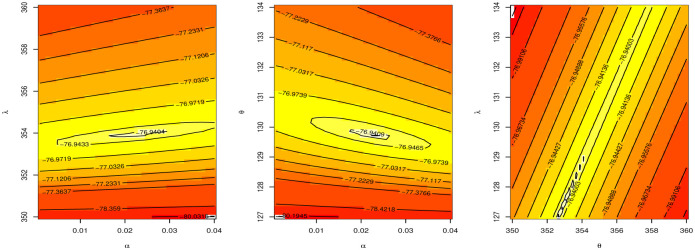
Contour plot of GAPL distribution for Netherlands COVID-19 mortality dates data.

**Fig 13:**
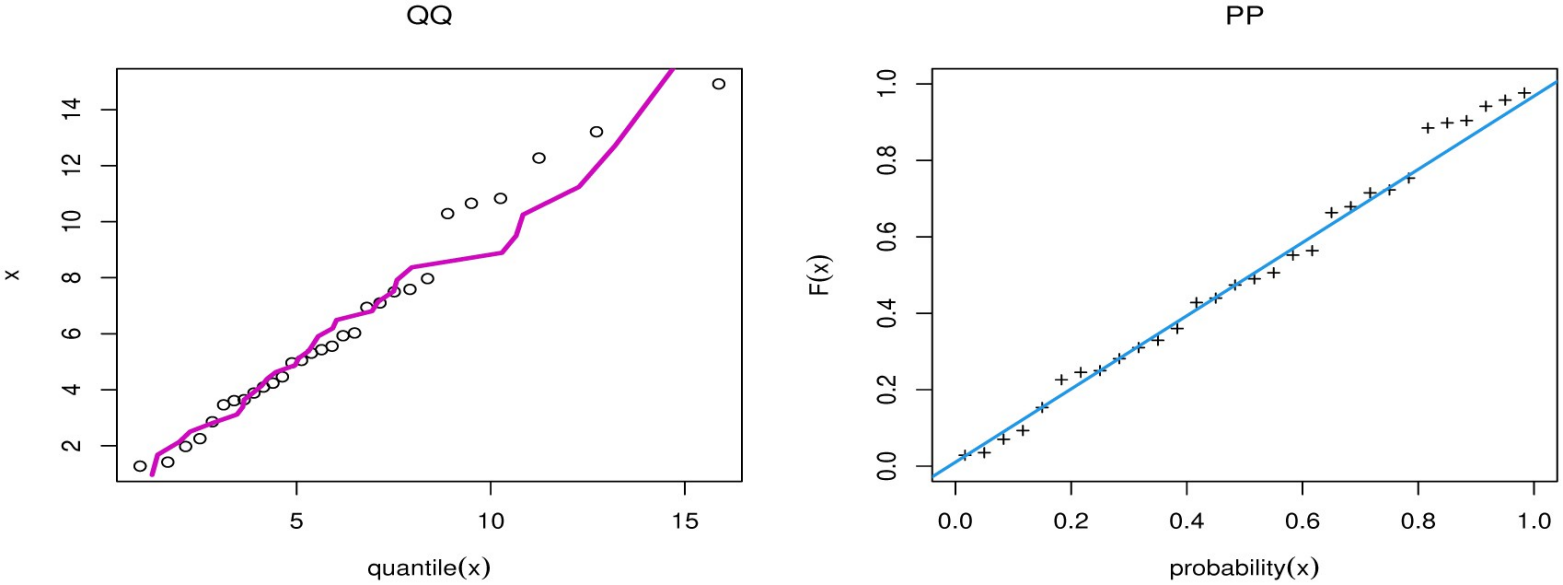
PP and QQ plots of GAPL distribution for Netherlands COVID-19 mortality dates data.

**Fig 14:**
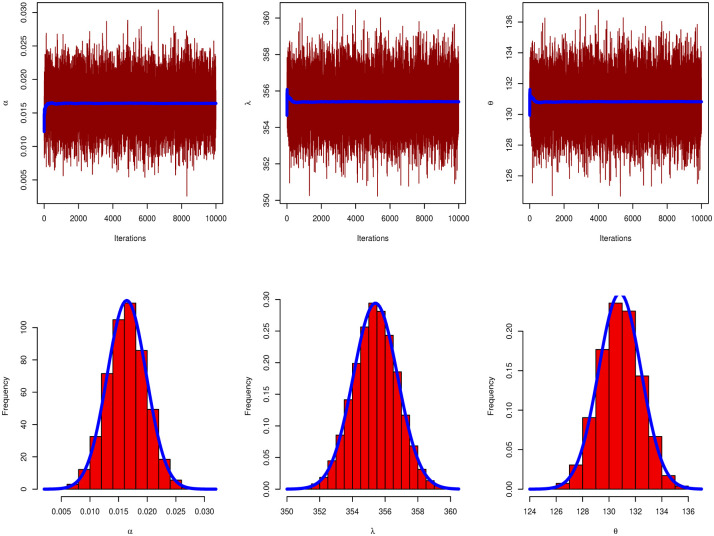
MCMC plot of GAPL distribution for Netherlands COVID-19 mortality dates data.

Fig 12 discussed the contour plot of GAPL distribution for the Netherlands COVID-19 mortality dates data. Also, Fig 13 shows the PP and QQ plots of GAPL distribution for the Netherlands COVID-19 mortality dates data. Table 13 discussed Bayesian estimation for parameters of GAPL for Netherlands COVID-19 mortality dates data. By comparing Bayesian and MLE in Table 11, we note that the Bayesian estimation has the smallest SE for parameters. Fig 14 shows MCMC plots of GAPL parameters for the Netherlands COVID-19 mortality dates data.

**Table 13:** Bayesian estimation for parameters of GAPL by Netherlands COVID-19 mortality dates data.

	*α*	λ	*θ*
estimates	0.0164	355.4074	130.8187
SE	0.0034	1.3567	1.6079

## 8 Conclusion

There will always be attempts to generalize distributions in distribution theory. The goal of generalizing distributions is to create more reliable, adaptable models with a variety of applications. Many techniques are used to do this, as numerous pieces of literature have shown. Additionally, the analysis and empirical findings heavily depend on how well the chosen distribution fits the input data. In this article, We suggested the Gull Alpha Power Lomax distribution as a new generalization of the Lomax distribution. The quantile and moments, among other statistical properties of this distribution, have been derived and discussed. The greatest likelihood estimators have been calculated. We performed a simulation study to compare these methods. We have compared estimators with respect to bias and mean-squared error. The simulation results revealed that the Bayesian method is a very competitive method among others. Compared with other distributions based on AIC, BIC, CAIC, HQIC, LL, KS, and probability values, the GAPL distribution proved stronger than the competing distributions. The new distribution is applied to two actual data sets that are related to the daily number of COVID-19 cases in China. The Lomax distribution, exponential Lomax, and power Lomax distributions are compared. We find that the proposed model is extremely competitive in terms of fitting this real data set based on the comparison criterion between all of these models.
